# Epidemiology of interstitial lung disease in patients with metastatic breast cancer at baseline and after treatment with HER2-directed therapy: a real-world data analysis

**DOI:** 10.1007/s10549-022-06738-6

**Published:** 2022-10-06

**Authors:** Jeanna Wallenta Law, Alicyn Campbell, Colin Weller, Colden Johanson, Ronda Broome, Elisabeth Piault, Monika Izano, Andrew Schrag, Mary Tran, Thomas D. Brown, Henry G. Kaplan

**Affiliations:** 1 Syapse, 303 2nd Street, North Tower, Suite 500, San Francisco, CA 94107 USA; 2 AstraZeneca, 1800 Concord Pike, Wilmington, DE 19850 USA; 3 Swedish Cancer Institute, 1221 Madison St, Seattle, WA 98104 USA

**Keywords:** Metastatic breast cancer, HER2-directed therapy, Interstitial lung disease, Real-world, Epidemiology

## Abstract

**Purpose:**

Using real-world data, interstitial lung disease (ILD) prevalence before and after HER2-directed therapy was estimated. Potential ILD risk factors in patients receiving HER2-directed therapy for metastatic breast cancer (mBC) were evaluated.

**Methods:**

Adults with HER2-directed therapy for mBC initiated between September 25, 1998, and February 22, 2020 were, included. ILD was defined broadly as one or more of 64 lung conditions. Patients were followed until incident ILD, death, last contact, or study end.

**Results:**

In total, 533 patients were identified with median age at mBC of 57, 51% had de novo mBC, 43% were ever smokers, 30% had lung metastases, 9% had thoracic radiation, 6% had chronic obstructive pulmonary disease, and 16% had prevalent ILD. ILD cumulative incidence at one year was 9% (95% CI 6%, 12%), with a median follow-up of 23 months. Smoking (HR 2.2, 95% CI 1.1, 4.8) and Black/African-American race (HR 3.4, 95% CI 1.6, 7.5) were significantly associated with ILD; HRs for preexisting lung conditions (HR 1.8, 95% CI 0.9, 3.8) and thoracic radiation (HR 2.3, 95% CI 0.8, 7.1) were not statistically significant. Prevalent ILD was associated with 13-fold greater occurrence of incident ILD. 85% of patients with prevalent or incident ILD were symptomatic.

**Conclusions:**

This real-world population of patients with mBC had a high prevalence of ILD prior to HER2-directed therapy, reflecting the multifactorial causation of interstitial lung changes. The cumulative incidence of ILD in patients receiving HER2-directed therapy for mBC augments prior reports. Symptomatic presentation suggests an opportunity for early intervention.

**Supplementary Information:**

The online version contains supplementary material available at 10.1007/s10549-022-06738-6.

## Introduction

Breast cancer is one of the most common cancers globally [1, 2], and in the USA, one in every eight women may have invasive breast cancer within her lifetime [2]. HER2-positive breast cancer is an aggressive subtype accounting for 15–20% of all cases [3]. Since the US Food and Drug Agency’s (FDA) 1998 approval of trastuzumab [4], additional HER2-directed therapies have been approved, and their use has dramatically improved progression-free and overall survival for patients with advanced HER2-positive breast cancer [5–9].

Along with their clinical benefits, many commonly used breast cancer treatments, including HER2-directed therapies, confer increased risk of treatment-related interstitial lung disease (ILD) [10–18]. ILD, a serious medical condition with multifactorial causation, is characterized by inflammation and fibrosis of the lung interstitium, often presenting with shortness of breath and/or cough or similar non-specific symptoms, and may be life-threatening in some patients [13, 16]. It is a diagnosis of exclusion, and there is no easily, systematically applied criteria [19–21]. Evaluation of the pulmonary toxicity of individual agents is challenging because breast cancer patients may have underlying lung conditions (metastatic disease or radiation-related), and often receive multiple therapeutic agents in varying doses, sequences, and combinations. The increased use of HER2-directed therapies, some with stated risk of ILD in their labels, underscores the need to identify factors that increase ILD risk to inform routine patient monitoring. Monitoring can inform timely ILD diagnosis and early intervention while maintaining patients on optimal HER2-directed therapies. This real-world retrospective study was conducted in patients with mBC to better understand the baseline prevalence of ILD, and the incidence and predictors of ILD associated with HER2-directed therapy, common ILD presentations, and its clinical management.

## Methods

### Study population

This retrospective study included patients from the Syapse Learning Health Network (LHN), a longitudinal database that captures data from cancer patients treated in community-based integrated care delivery networks across 25 states in the USA, including 457 hospitals and over 1300 oncologists. Each patient’s records include data from various sources: electronic health records (EHR), laboratory and radiology/imaging systems, computerized order entry systems, and hospital-based cancer registries. The study population included all patients who were 18 years or older at time of initial breast cancer diagnosis, had evidence of mBC (stage IV at diagnosis or physician-assessed progression/recurrence), and had initiated a HER2-directed therapy in the metastatic setting between September 25, 1998, and February 22, 2020. Patients with locally advanced disease who had not developed metastases were not included in this study. Patients were followed from their index date (initiation of HER2-directed therapy in the metastatic setting) until the earliest of their first incident ILD event (defined below) or date of death, last contact, or study end on May 22, 2020. This study end date allowed for a minimum of three months of potential follow-up when data analysis and curation started. The date of death was determined by a validated composite mortality score [22].

### HER2-directed therapy assessment

To allow for six months capture of drug prescribing in routine care, the following HER2-directed therapies, all approved by the US FDA prior to December 2019, were included: trastuzumab, trastuzumab-anns, trastuzumab-dkst, trastuzumab-dttb, trastuzumab-pkrb, trastuzumab-qyyp, ado-trastuzumab emtansine, neratinib, lapatinib, and pertuzumab. Patients treated with trastuzumab deruxtecan or tucatinib during the study period were excluded as these agents were approved after December 2019.

### ILD assessment

ILD was defined by the presence of at least one of 64 lung conditions identified through review of clinician notes, imaging narratives, pathology reports, bronchoscopy results, and relevant International Classification of Diseases (ICD) codes recorded in patient charts (Supplemental Table 1). Chronic obstructive pulmonary disease (COPD), while documented separately, was excluded from the list of terms defining ILD. Given the lack of a functional definition of ILD, in an attempt to include all the relevant terms and case presentations in this analysis, the standardized Medical Dictionary for Regulatory Activities (MedDRA) queries for ILD were used and augmented with any term considered to be indicative of a potential pulmonary inflammatory or fibrotic event based on expert clinical input (Supplemental Table 1). This broad operational definition of ILD was used to capture any possible occurrence of interstitial lung changes, document the heterogeneous nature of ILD, and capture the earliest signs of the ILD. To reduce potential misclassification, records for patients with pleural effusion alone on or after the patient’s diagnosis of mBC were reviewed in detail, and patients with evidence of malignant pleural effusion unaccompanied by a second qualifying lung condition were not considered to have ILD. Patients with lung metastases who did not present with pleural effusion or other ILD lung conditions were also not considered to have ILD. Patients with ILD identified exclusively in the follow-up (post-index) period were classified as having incident ILD. Patients with any evidence of ILD in the baseline period were considered to have prevalent ILD.

### Covariates

HER2-directed therapy use in the neoadjuvant and adjuvant settings, use of potentially ILD-inducing therapies (Supplemental Table 2) [16], and radiation to the thoracic region were assessed in the one-year pre-index period. Age, race, and performance status were documented at metastatic disease diagnosis date, and body mass index (BMI) was assessed at index date. Performance status measures were mapped to Eastern Cooperative Oncology Group (ECOG) performance status if alternative performance measures were documented. Smoking history was assessed at the initial breast cancer diagnosis date and each patient was categorized as an “ever” or “never” smoker. Additionally, pre-index data were used to assess baseline patient demographic and clinical characteristics, including identifying prevalent ILD and the presence of associated preexisting lung conditions such as COPD, pulmonary embolism, lung cancer, or lung metastasis. “Rapid” initiation of HER2-directed therapy was defined as starting HER2-directed therapy within 1 month from the date of first metastasis.

Relevant clinical attributes were extracted from the EHRs of patients with evidence of ILD. These data included: occurrence of ILD-specific lung diagnosis, signs and symptoms and their timing, and anticancer treatment regimen at ILD onset. In addition, an interval spanning six months before the first recorded ILD event until six months after the last recorded ILD event was used to search for hospitalizations, emergency room (ER) visits, supplemental oxygen, and mechanical ventilation. The use of steroid treatment for ILD management was also assessed.

### Statistical analysis

The distribution of baseline patient demographic and clinical characteristics was evaluated for the population overall and additionally stratified by ILD status per three mutually exclusive groups: (1) the “none” group includes patients without prevalent or incident ILD; (2) the “prevalent” group includes patients with prevalent ILD, excluding those with incident ILD; and (3) the “incident” group includes patients with incident ILD, excluding those with prevalent ILD. The distributions of categorical variables were summarized as frequencies and percentages, while medians and interquartile ranges (IQR: 25th percentile, 75th percentile) were reported for continuous variables.

Cumulative incidence curves were used to characterize the distribution of time to the first incident ILD event for the subset of patients without prevalent ILD. Patients who were diagnosed with mBC in or after 2010, a time period in which death data were available, were considered for all time-to-event analyses. Results were reported separately for ILD incidence for two scenarios: *anytime after* the initiation of HER2-directed therapy and ILD incidence *during* HER2-directed therapy. In the first scenario, patients were followed for incident ILD from index date to the earliest of first recorded ILD event, death, last contact, or end of study.

To account for variable duration of HER2-directed therapy in the second scenario, patients were followed from their index date to the earliest of first recorded ILD, first HER2-directed therapy discontinuation or termination, date of death, date of last contact or study end. Gaps in HER2-directed therapy of 30 days or less were bridged, creating a contiguous treatment period. Cumulative incidence estimates are reported overall, and additionally stratified by smoking status and presence/absence of associated preexisting lung conditions. Additionally, Cox proportional hazard models were used to estimate associations between pre-index demographic and clinical factors and incident ILD risk while on HER2-directed therapy. These associations were assessed among all patients, as well as restricted to those without prevalent ILD, in the event that subsequent ILD may reflect unresolved, existing disease in patients with prevalent ILD. Models included the following covariates: age, race, body mass index (BMI), smoking status, associated preexisting lung conditions, history of thoracic radiation excluding radiation to intact breast, potentially ILD-inducing systemic therapy, time between mBC diagnosis and index date, ECOG performance score, and presence/absence of prevalent ILD. The proportional hazards assumption was assessed graphically using Schoenfeld residuals.

All data analyses were performed in R version 3.6.1. The level of significance was set at 0.05.

## Results

### Study population characteristics

From among 2,490 patients with mBC, 533 (including 7 males) met the selection criteria for study inclusion (Table 1). The majority of patients were white (77%) and the median age at diagnosis of mBC was 57 years (IQR: 48, 65 years); 51% had de novo metastatic disease (Table 2). Approximately 43% of patients had a history of smoking (ever smoked) at the time of their initial breast cancer diagnosis and 30% had lung metastasis.

**Table 1:** Attrition

Criterion	Description	*N* included	*N* excluded
Breast cancer patients with CTR-curated data availability	Syapse identifies potentially eligible patients via our structured data sources via International Classification of Disease diagnosis codes (ICD9 174.x or 175.x or ICD10 C50.x) Patient’s histology confirmed disease is CTR-verified prior to curation	15,689	
Known dates of breast cancer diagnosis	Has breast cancer diagnosis date	14,383	1306
Age 18 years or older	Patient is 18 or older at breast cancer diagnosis	14,382	1
Evidence of mBC diagnosis	mBC - stage at diagnosis IV, IVa, IVb, IVc - or clinician assessed patient has progressed to metastatic disease	2490	11,892
Exposure to HER2-directed therapy in mBC setting by February 22, 2020^a^	HER2-directed therapies approved prior to December 2019: HER2-directed therapies include: Trastuzumab Pertuzumab Ado-Trastuzumab emtansine Neratinib Lapatinib Trastuzumab-anns Trastuzumab-dkst Trastuzumab-dttb Trastuzumab-pkrb Trastuzumab-qyyp	544	1946
No exposure to trastuzumab deruxtecan prior to study end date (May 22, 2020)		533	11

*CTR* certified tumor registrar, *HER2* human epidermal growth factor receptor 2, *ICD* International classification of diseases, *mBC* metastatic breast cancer

^a^Index date defined by first exposure. This end date allows patients to have a minimum of 3 months of potential post-index follow-up prior to study end date

**Table 2:** Baseline demographics and clinical characteristics,^a^ stratified by ILD status

	No ILD (*N* = 384)	Prevalent ILD (*N* = 84)	Incident ILD (*N* = 65)	Total (*N* = 533)
Age at initial breast cancer diagnosis [median (IQR)]	53 (44, 62)	58 (46, 66)	54 (45, 62)	53 (45, 63)
Age at metastatic diagnosis [median (IQR)]	56 (47, 64)	61 (52, 69)	57 (48, 64)	57 (48, 65)
Race [*n* (%)]				
White	298 (78)	64 (76)	48 (74)	410 (77)
Black or African-American	67 (17)	14 (17)	17 (26)	98 (18)
Asian	11 (3)	3 (3.5)	0 (0)	14 (3)
Other or not provided	8 (2)	3 (3.5)	0 (0)	11 (2)
Ethnicity				
Hispanic/Latino	22 (6)	4 (5)	1 (2)	27 (5)
Non-Hispanic/Non-Latino	357 (93)	76 (90)	62 (95)	495 (77)
Not provided	5 (1)	4 (5)	2 (3)	11 (2)
Stage IV at diagnosis [*n* (%)]	193 (50)	39 (46)	38 (58)	270 (51)
ECOG at metastatic diagnosis [*n* (%)]				
0	68 (18)	19 (23)	12 (18)	99 (19)
1	61 (16)	16 (19)	7 (11)	84 (16)
2+	26 (7)	8 (9)	5 (8)	39 (7)
Unknown	229 (60)	41 (49)	41 (63)	311 (58)
BMI				
Normal	95 (25)	24 (29)	19 (29)	138 (26)
Overweight	102 (27)	22 (26)	10 (15)	134 (25)
Obese	118 (31)	31 (37)	21 (32)	170 (32)
Unknown	69 (18)	7 (8)	15 (23)	91 (17)
Menopausal status at metastatic diagnosis				
Premenopausal	91 (24)	10 (12)	16 (25)	117 (22)
Perimenopausal	8 (2)	1 (1)	1 (2)	10 (2)
Postmenopausal	240 (62)	65 (77)	36 (55)	341 (64)
Unknown/not available	45 (12)	8 (9)	12 (18)	65 (12)
Smoking history at initial diagnosis (ever smoked)	157 (41)	37 (44)	34 (52)	228 (43)
Thoracic radiation^b^	35 (9)	8 (10)	6 (9)	49 (9)
Lung metastasis	98 (26)	37 (44)	23 (35)	158 (30)
COPD	14 (4)	9 (11)	7 (11)	30 (6)
Pulmonary embolism	5 (1)	5 (6)	0 (0)	10 (2)
Lung cancer	1 (0)	2 (2)	0 (0)	3 (1)
Anemia	93 (24)	33 (39)	13 (20)	139 (26)
Autoimmune condition^c^	12 (3)	4 (5)	2 (3)	18 (3)

*BMI* Body mass index, *COPD* chronic obstructive pulmonary disease, *ECOG* Eastern Collaborative Oncology Group, *ILD* interstitial lung disease, *IQR* interquartile range, *n*: number

^a^Assessed at index date unless otherwise specified

^b^Definitive, palliative or prophylactic radiation to lungs, mediastinal, and chest wall for lung metastasis from breast cancer, lung cancer, or treatment that included lung in the therapy field. Does not include radiation received to the intact breast

^c^Lupus, rheumatoid arthritis, sarcoidosis, scleroderma, and/or polymyositis/dermatomyositis

The majority of our study population had received a suspected ILD-inducing treatment prior to the metastatic setting. Approximately one in four patients (23%, *n* = 120) had received HER2-directed therapy in the neoadjuvant or adjuvant setting, with the majority (77%, *n* = 92) receiving HER2-directed therapy for more than three months, and 33% continuing for longer than one year. Half of the participants had completed neoadjuvant or adjuvant HER2-directed therapy 12 or more months prior to index, and 23% were on HER2-directed therapy when metastatic disease was detected. One in three patients (32%, *n* = 171) received another (non-HER2-directed) potentially ILD-inducing therapy prior to index therapy, with taxanes being the most common of such. The majority of these patients had more than three months of prior potentially ILD-inducing therapy, and most (60%, *n* = 102) discontinued such at least one year prior to index therapy.

### Prevalent and incident ILD presentation and management

Approximately 16% of patients had prevalent ILD at index. Patients with prevalent ILD and incident ILD had a similar burden of COPD (11%) and the proportions of patients who received thoracic radiation were similar across the incident ILD, prevalent ILD, and none (neither prevalent nor incident ILD) groups. While smoking proportion was higher in the incident group, anemia and lung metastasis were more common in the none and prevalent groups. Among patients with incident ILD, 55% were on a HER2-directed therapy in combination with a different ILD-inducing treatment at ILD onset, while 39% were only on a HER2-directed therapy (Table 2). 97% of ILD lung conditions were found via high-resolution chest CT scan image narratives. Pleural effusion was the most common ILD lung condition identified in both the prevalent and incident ILD groups, followed by nodular opacities, pneumonia, and ground glass opacities. Presentations of disease varied with 9% of patients presenting with only pleural effusion (prevalent: 12%; incident: 6%) and 24% presenting with more than 5 ILD lung conditions over time (prevalent: 21%; incident: 28%). Over 80% of patients with prevalent or incident ILD were reported as either symptomatic at onset or moved from an asymptomatic state to symptomatic during the study. Shortness of breath/dyspnea was the most common symptom at presentation for both prevalent and incident ILD, followed by cough. A minority of patients received steroids for ILD (prevalent: 20%; incident: 14%), with a median time between the first ILD indication and steroid initiation of 2.3 months for patients in the prevalent ILD group and 8.6 months for those in the incident ILD group. Respiratory-related hospital admissions and ER visits were more common in the prevalent ILD group compared with the incident ILD group, but the opposite was true for the use of supplemental oxygen. The proportion of patients in each ILD group who required mechanical ventilation was similar (prevalent: 7%; incident: 6%) (Table 3).

**Table 3:** Characteristics of ILD presentation among patients with prevalent or incident ILD

	Prevalent ILD (*N* = 84)	Incident ILD (*N* = 65)
ILD lung conditions in follow-up period [*n* (%)]		
Pleural effusion	65 (77)	35 (54)
Nodular opacities	32 (38)	29 (45)
Pneumonia	27 (32)	27 (42)
Ground glass opacities	20 (24)	26 (40)
Pleural effusion only	10 (12)	4 (6)
Number of ILD lung conditions in follow-up period [*n* (%)]		
1	19 (23)	14 (22)
2	23 (27)	14 (22)
3	18 (21)	10 (15)
4	6 (7)	9 (14)
5+	18 (21)	18 (28)
Symptom timing [*n* (%)]		
Began symptomatic	52 (62)	43 (66)
Asymptomatic throughout	13 (15)	10 (15)
Symptoms presentation [*n* (%)]		
Shortness of breath	50 (60)	43 (66)
Cough	24 (29)	25 (38)
Chest pain	22 (26)	6 (9)
Hypoxia	3 (4)	10 (15)
Other	27 (32)	18 (28)
Steroids		
Treated with steroids [*n* (%)]	17 (20)	9 (14)
Time to treatment (months) [median (IQR)]	2.3 (1, 6.9)	8.6 (0.2, 12.1)
Length of treatment (months) [median (IQR)]	1 (0.2, 3.7)	2.5 (1.1, 4.6)
ER visits	(36)	(31)
Hospitalizations		
Admitted to the hospital [n (%)]	(75)	(78)
Length of admission (days) [median (IQR)]	6 (3, 9)	6 (3, 10)
Respiratory-related admission reason [n (%)]	(45)	(36)
Ventilator support	6 (7)	4 (6)
Supplemental oxygen	35 (42)	32 (49)

*ILD* interstitial lung disease, *IQR* interquartile range, *n* number

### Cumulative incidence of ILD and associated factors

**Fig. 1:**
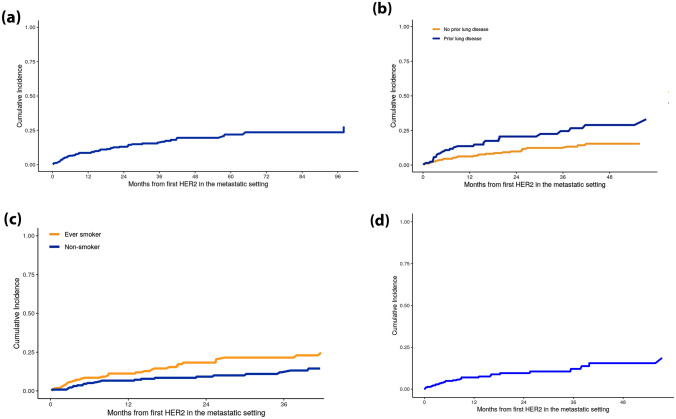
**a** Cumulative incidence of ILD among patients without prevalent ILD at index (*n* = 398). **b** Cumulative incidence of ILD among patients without prevalent ILD at index (*n* = 398), stratified by prior lung disease*. **c** Cumulative incidence of ILD among patients without prevalent ILD at index (*n* = 398), stratified by smoking history. **d** Cumulative incidence of ILD while on HER2-directed therapy** among patients without prevalent ILD at index (*n* = 398) *Lung disease: prior COPD, pulmonary embolism, lung cancer, or lung metastasis at first HER2 directed therapy in metastatic setting. Note this definition does not include any ILD keywords, therefore is mutually exclusive from the outcome of ILD. **“While on HER2 directed therapy” was defined as from the first HER2 directed therapy to the end of the first HER2 directed therapy, allowing for 30 day gaps in treatment

The cumulative incidence of ILD following initiation of HER2-directed therapy in the metastatic setting was assessed for 398 patients without prevalent ILD at index whose metastatic disease was diagnosed in 2010 or later. The median follow-up for these patients was 23 months. The cumulative incidence of ILD at one year was 9% (95% confidence interval [CI]: 6%, 12%, Fig. 1a). The cumulative incidence of ILD at one year was greater among patients with associated preexisting lung conditions than among patients without (14% vs. 6%, Fig. 1b), and also higher among ever smokers than among never smokers (11% vs. 6%, Fig. 1c). The estimated cumulative incidence of ILD at one year while on HER2-directed therapy was 7% (95%CI: 5%, 11%) (Fig. 1d).

In the Cox proportional hazard analyses that excluded patients with prevalent ILD (*n* = 398), smoking (HR 2.2, 95% CI 1.1, 4.8) and Black/African-American race (HR 3.4, 95% CI 1.6, 7.5) were significantly associated with increases in the hazard ratio of incident ILD; associated preexisting lung conditions (HR 1.8, 95% CI 0.9, 3.8) and thoracic radiation (HR 2.3, 95% CI 0.8, 7.1) were associated with increases in the hazard ratio that did not achieve statistical significance (Table 4).

**Table 4:** Cox regression of incident ILD while on first HER2-directed therapy, *n* = 479

Variable	*P* value	HR (95% CI)	*N*	*N* events
Age > 65 years	0.716	1.1 (0.7, 1.8)	123	24
Black/African-American	0.12	1.5 (0.9, 2.6)	93	20
BMI: Overweight (25–30)	0.221	0.7 (0.4, 1.3)	126	17
BMI: Obese (30+)	0.797	0.9 (0.6, 1.6)	167	31
Ever smoker	0.543	1.2 (0.7, 1.8)	204	38
Prior lung comorbidities	0.099	1.9 (0.9, 3.8)	276	67
Prior thoracic radiation	0.168	1.7 (0.8, 3.5)	38	9
Prior ILD-inducing systemic therapy	0.544	0.9 (0.5, 1.4)	156	24
< 1 month from mBC to index	0.143	1.4 (0.9, 2.2)	222	40
ECOG: > 0	0.063	1.9 (1, 3.6)	121	26
Has prevalent ILD at index	< 0.001	13 (7.4, 22.7)	81	48

*BMI* body mass index, *CI* confidence interval, *ECOG* Eastern Collaborative Oncology Group, *HR* hazard ratio, *ILD* interstitial lung disease, *mBC* metastatic breast cancer, *N* number

## Discussion

The incidence of ILD in this cohort of patients who received HER2-directed therapies for mBC in the US routine care setting was 9% at one year. This estimate is in line with previous reports that range from 2.4 to 21% [13]. Cumulative incidence of incident ILD at 1 year was higher among patients with preexisting lung conditions and smokers, compared to patients without associated preexisting lung conditions and non-smokers.

In Cox models that included prevalent ILD cases, prevalent ILD was the strongest predictor of incident ILD, with an estimated 13-fold increase in risk. Other preexisting lung conditions, and thoracic irradiation, also appeared to increase the risk. While it is unclear whether incident ILD events were truly new events or simply reflected existing disease in patients with prevalent ILD, our findings from analyses that excluded these patients indicate that Black/African-American race and history of smoking are associated with increased risk of incident ILD after the initiation of HER2-directed therapies in the metastatic treatment setting. These insights contribute to a more complete understanding of the “at-risk” population, with the potential to improve treatment and outcomes.

Certain limitations should be considered while interpreting the results of this study. It is possible that the available baseline data do not completely capture all relevant pre-index comorbidity. Baseline characteristics, including prevalent lung comorbidities, may be less accurately captured for patients who entered the integrated care delivery system shortly prior to the initiation of HER2-directed therapy. There is potential for misclassifying patients as having incident ILD; any missed cases would likely be lower grade as more severe cases would generally require medical attention and be well-captured in the health system records. Additionally, it is possible that some incident events may reflect unreported prevalent disease. It is possible that the exclusion of associated lung conditions (COPD, pulmonary embolism, lung cancer, or lung metastasis) from our definition of ILD may have contributed to further outcome misclassification; however, this risk was mitigated by the inclusion of these conditions as risk factors in the multivariate models. In sensitivity analyses that included prevalent ILD cases (*n* = 479), prevalent ILD was the only factor statistically significantly associated with ILD, increasing the hazard of subsequent ILD by 13-fold (95% CI 7.4, 22.7; Table 5); this suggests that prevalent disease tends to persist during follow-up. While we accounted for duration of HER2-directed therapy in the metastatic setting by allowing it to determine length of follow-up in a subset of our time-to-event analyses, dosage during follow-up, duration of HER2-directed therapy in the neoadjuvant or adjuvant settings, dosing and duration of thoracic radiation, and dosing and duration of other ILD-inducing medication use were not evaluated in this analysis. Bridging of up to 30-day gaps in HER2-directed therapy treatment may have misclassified exposure for some patients, although bridging of multiple gaps during treatment journey was uncommon in our analysis. In light of increasing evidence for a possible link between HER2-directed therapies and ILD in multiple tumor types, it is possible that patients with HER2-positive mBC at high risk for ILD were treated at lower doses or received closer monitoring than lower-risk patients which could lead to underestimation of the true incidence of treatment-related ILD in high-risk patients. Of additional note, the group of patients without prevalent ILD, i.e., the “incident” group, from which the cumulative incidence of ILD at one year was determined, had a modest median follow-up of 23 months. Lastly, due to the timeframe of the study, at least one, more recently approved HER2-directed therapy is not included in this analysis. Understanding the epidemiology, diagnosis, and management of ILD in patients treated with all available HER2-directed therapies will become increasingly important in the coming years.

**Table 5:** Cox regression of incident ILD while on first HER2-directed therapy among patients without prevalent ILD at index, *n* = 398

Variable	P-value	HR (95% CI)	*N*	*N* events
Age > 65 years	0.926	1 (0.4, 2.3)	94	7
Black/African-American	0.002	3.4 (1.6, 7.5)	79	12
BMI: overweight (25–30)	0.274	0.5 (0.2, 1.6)	105	6
BMI: obese (30+)	0.783	0.9 (0.4, 2.2)	137	12
Ever smoker	0.037	2.2 (1.1, 4.8)	168	17
Prior lung comorbidities	0.124	1.8 (0.9, 3.8)	195	19
Prior thoracic radiation	0.143	2.3 (0.8, 7.1)	30	4
Prior ILD-inducing systemic therapies	0.145	0.5 (0.2, 1.3)	122	5
Less than 1 month from first metastasis to index	0.185	1.7 (0.8, 3.5)	183	16
ECOG: > 0	0.266	1.8 (0.6, 5.1)	97	9

*BMI* body mass index, *CI* confidence interval, *ECOG* Eastern Collaborative Oncology Group, *HR* hazard ratio, *ILD* interstitial lung disease, *N* number

This study also has a number of strengths. Access to both oncology and non-oncology data enables more complete assessment of both baseline comorbidities as well as ILD conditions. The broad definition of ILD also enabled identification of the earliest indications of interstitial lung changes and supports the inclusion of diverse presentations of ILD. Importantly, these data show what is happening in routine clinical care. Real-world evidence provides an opportunity to study the tolerability of drug regimens in routine clinical practice serving a varied patient population that may not be eligible for enrollment in clinical trials. Real-world evidence also delivers a complementary view to learnings from randomized controlled trials.

To our knowledge, this study provides the first and most detailed evaluation of the pre-treatment prevalence, and subsequent incidence of ILD, along with associated risk factors, in a real-world, and thus clinically diverse, patient population with mBC who received HER2-directed therapies within US community health systems. To date, most reports of treatment-related ILD in breast cancer patients receiving HER2-directed therapies reflect the experience in the clinical trial setting [13]. Future research should build on this study of ILD to improve supportive care for patients responding well to HER2-directed therapies, thus maximizing therapeutic benefit.

## Supplementary Information

Below is the link to the electronic supplementary material.

Supplementary file1 (DOCX 20 kb)

## Data Availability

The datasets generated and analyzed for the current study are proprietary and therefore, not publicly available.
