# Supplementary material for: Epidemiology of interstitial lung disease in patients with metastatic breast cancer at baseline and after treatment with HER2-directed therapy: a real-world data analysis
Source: Breast Cancer Res Treat. 2022 Oct 6;196(3):603–11. doi: 10.1007/s10549-022-06738-6 (PMC9633512; doi:10.1007/s10549-022-06738-6)
Supplement: Supplementary file 1 — Supplementary file1 (DOCX 20 kb) [file 10549_2022_6738_MOESM1_ESM.docx]

**Supplemental Table 1. Lung conditions used to identify ILD**

| Acute interstitial pneumonitis |
| --- |
| Acute respiratory distress syndrome |
| Acute respiratory failure |
| Air-space consolidation |
| Allergic eosinophilia |
| Alveolar lung disease |
| Alveolar proteinosis |
| Alveolitis |
| Alveolitis allergic |
| Alveolitis necrotizing |
| Architectural distortion |
| Autoimmune lung disease |
| Bronchiolitis |
| Chronic interstitial pneumonia (CIP) |
| Combined pulmonary fibrosis and emphysema |
| Diffuse alveolar damage |
| Drug-induced interstitial lung disorder |
| Eosinophilia myalgia syndrome |
| Eosinophilic granulomatosis with polyangiitis |
| Eosinophilic pneumonia |
| Eosinophilic pneumonia acute |
| Eosinophilic pneumonia chronic |
| Granulomatous pneumonitis |
| Ground-glass attenuation |
| Idiopathic interstitial pneumonia |
| Idiopathic pneumonia syndrome |
| Idiopathic pulmonary Fibrosis |
| ILD |
| Interlobular septal thickening |
| Interstitial lung disease |
| Interstitial pneumonia |
| Interstitial pulmonary disease |
| Intralobular reticular opacity |
| Lung infiltration |
| Lymphangitic carcinomatosis |
| Necrotising bronchiolitis |
| Nodular opacities |
| Non-septal linear opacity |
| Obliterative bronchiolitis |
| Organising pneumonia |
| Pleural effusion |
| Pneumonitis |
| Previous tuberculosis |
| Progressive massive fibrosis |
| Pulmonary emphysema |
| Pulmonary fibrosis |
| Pulmonary necrosis |
| Pulmonary radiation injury |
| Pulmonary sarcoidosis |
| Pulmonary toxicity |
| Pulmonary vasculitis |
| Radiation alveolitis |
| Radiation fibrosis |
| Radiation pneumonitis |
| Respiratory failure |
| Restrictive pulmonary disease |
| Rheumatoid lung |
| Sarcoidosis |
| Scarring or inflammation of interstitium |
| Small airways disease |
| Thickening of bronchovascular bundles |
| Traction bronchiectasis |
| Transfusion-related acute lung injury |
| Pneumonia |

**Supplemental Table 2. Other potentially ILD inducing drugs**

| Abatacept |
| --- |
| Adalimumab |
| Afatinib |
| Amiodarone |
| Atezolizumab |
| Avelumab |
| Bleomycin |
| Cemiplimab |
| Certolizumab |
| Certolizumab-pegol |
| Docetaxel |
| Durvalumab |
| Erlotinib |
| Etanercept |
| Everolimus |
| Fluorouracil, epirubicin and cyclophosphamide (FEC) followed by weekly paclitaxel |
| Gefitinib |
| Gemcitabine |
| Gemcitabine + Vinorelbine |
| Golimumab |
| Infliximab |
| Ipilimumab |
| Leflunomide |
| Methotrexate |
| Nab-paclitaxel |
| Nitrofurantoin |
| Nivolumab |
| Osimertinib |
| Paclitaxel |
| Pembrolizumab |
| Rituximab |
| Sirolimus |
| Temsirolimus |
| Tocilizumab |

ILD: interstitial lung disease
